# Laparoscopic Splenectomy for Hairy Cell Leukemia in Pregnancy

**DOI:** 10.1155/2010/136823

**Published:** 2010-09-16

**Authors:** Beni Adegoke Adeniji, Moses Fallas, Marc Incerpi, Solomon Hamburg, Robert Katz, Dotun Ogunyemi

**Affiliations:** ^1^Cedars-Sinai Medical Center, 8700 W Beverly Bowerard, Los Angeles, CA 90048, USA; ^2^LAC+USC Medical Center, 1420 S Central Avenue, Glendale, CA 91201, USA

## Abstract

*Objective*. We present a successful case of laparoscopic splenectomy for a massively enlarged spleen at 25 weeks of gestation for hairy cell leukemia in pregnancy in a woman with initial hemoglobin of 4.3 gm/dl and platelet count of 18,000/mm^3^. *Study Design*. Case report. *Results*. This report provides an approach to management that may be applicable in those cases where thrombocytopenia or other clinical imperatives preclude delaying treatment till after pregnancy. *Conclusion*. Hairy cell leukemia is a clonal B-Cell malignancy, for which there is very limited experience worldwide for its management when it occurs during pregnancy. Laparoscopic splenectomy should be considered as a therapeutic option, even with a significantly enlarged spleen, in order to avoid the risks of fetal exposure to chemotherapeutic agents. Unique considerations relating to pregnancy are highlighted.

## 1. Introduction

Hairy cell leukemia (HCL) is a chronic lymphoid leukemia recognized as a clonal B-cell malignancy. It presents with abnormal cells which have hairlike cytoplasmic projections on the surface (under light microscopy) by which the disease was named in 1966 by Schrek and Donnelly [1]. It is a rare form of leukemia, with an incidence rate of 0.33 per 100,000 person years in the United States [2]. The mean age at presentation is 52 years, with a male Caucasian preponderance. Historically treated by splenectomy and chemotherapy with single agent 2-chlorodeoxyadenosine (Cladribine, 2-CdA) being currently the preferred treatment because of its efficacy whilst avoiding the risks of major surgery. HCL is even more uncommon in pregnancy, with a review of worldwide literature documenting only six (6) prior cases of HCL in pregnancy [3, 4].

## 2. Case Report

Our patient is a 37-year-old gravida 2 para 1 Caucasian female who presented at 23 weeks gestation with recurrent epistaxis and ecchymosis of the soft palate. Clinical evaluation revealed pancytopenia and splenomegaly. On her initial blood count, white cell count was 900/mm^3^, hematocrit 13.7%, and platelet count, 18,000/mm^3^. Bone marrow biopsy was consistent with HCL, CD 20 positive, CD 68 positive, and DBA 44 positive. The spleen measured approximately 17 × 13 × 4.5 cm on abdominal sonography. The patient desired continuation of pregnancy and was unwilling to accept the risks of chemotherapy or interferon alpha treatment in pregnancy. Treatment with Prednisone (60 mg per day) was initiated but the hematologic indices deteriorated further. Following extensive multidisciplinary consultations, it was decided that laparoscopic splenectomy offered the best potential for sustained improvement in hematologic indices whilst allowing the pregnancy to progress to viability with the least fetal risk. The patient was extensively counseled regarding the risks and benefits in line with the guidelines for use of laparoscopy for surgical problems during pregnancy as recommended by the Society of American Gastrointestinal and Endosopic Surgeons (SAGES) [5]. She received the polyvalent pneumococcal vaccine prior to splenectomy.

A laparoscopic splenectomy was performed at 25 weeks gestation. The patient was admitted one day before the proposed surgery for transfusion with packed red blood cells and platelets were transfused just prior to surgery. Her preoperative platelet count was 46,000/mm^3^ and hematocrit 29.5%. Adequate perioperative hydration was ensured and fetal heart rate monitoring was reassuring (continuous monitoring was suspended after induction of general anesthesia). The patient was placed in supine position with a left lateral tilt and general anesthesia with endotracheal intubation was administered. A direct incision was made in the midline, above the umbilicus just cephalad to the fundus of the uterus, as identified by palpation. A 5 mm sleeve was placed under direct visualization followed by establishment of carbon dioxide pneumoperitoneum at insufflation pressures of 10–15 mmHg. The spleen and other intraabdominal organs were visualized. The 5 mm trocar was then replaced with a 10–12 mm port and three additional trocars placed under direct laparoscopic visualization. Using a combination of harmonic scalpel and endo GIA stapler, the spleen was isolated in the usual fashion (sequentially mobilizing the splenorenal and splenophrenic ligaments, stapling hilar and short gastric vessels and finally releasing the superior splenophrenic attachments to free the spleen completely). It was then placed into an extra large Cook bag. The open end of the bag was exteriorized through the 12 mm port and the spleen was morcellated and removed with the bag. The spleen weighed 500 gms, consisted of mostly red pulp and hairy cell leukemia was confirmed on histopathological examination. The patient had an uneventful postoperative course.

She was discharged home after 2 days in hospital, the blood counts stabilized, and she was seen weekly for blood counts and clinical assessments. Serial fetal ultrasound scans showed normal growth trajectory. At 34 weeks gestation, the platelet count was back down to 32,000/mm^3^ and hematocrit of 22%. The decision was made to proceed with delivery of the infant in order to commence chemotherapy for the patient. Betamethasone was given for fetal lung maturation. Blood and platelet transfusion to hematocrit of 31% and platelet count of 100,000 was accomplished. Induction of labor with intracervical foley balloon and pitocin resulted in the delivery of a female infant with birth weight 2031 gms and Apgar scores 8 and 9 at 1 and 5 minutes, respectively. Histopathologic examination of the placenta showed slightly immature but otherwise normal 3rd trimester features without evidence of malignancy or leukemic infiltration. 

The vaginal delivery was accomplished without incident. However, within A few hours postpartum, she developed epigastric pain of increasing severity and retching without emesis. Esopagogastroduedonoscopy (EGD) showed Mallory-Weiss tears at the distal esophagus without leak. CT with contrast showed diaphragmatic hernia on the left side, and the patient underwent emergency laparoscopy followed by laparotomy at which the incarcerated stomach was reduced and a 4 cm rent in the diaphragm was closed. The rent was located approximately 2 cm to the left of the esophageal hiatus.

Chemotherapy for the hairy cell leukemia was commenced about six(6) weeks postop and she received a standard course of 5 cycles of Cladribine (0.12 mg/Kg). She is currently being followed in the oncology clinic and remains asymptomatic with normal hematologic indices. Her infant had attained the appropriate developmental milestones at six months of age.

## 3. Comment

Our case confirms the feasibility of laparoscopic splenectomy late in the second trimester even for a significantly enlarged spleen as may occur with hairy cell leukemia. It is possible that the splenectomy may have predisposed to a weakness in the diaphragmatic musculature or perhaps exposed an intrinsic weakness resulting in the gastric incarceration due to diaphragmatic herniation. This uncommon complication may be especially likely in the setting of the strong valsalva efforts that could have occurred as a consequence of the vaginal delivery along with diaphragmatic smooth muscle relaxation from elevated progesterone levels in pregnancy. 

The possibility of iatrogenic damage to the diaphragm during the laparoscopic splenectomy cannot be completely excluded. Although the surgeons involved in this case are advanced laparoscopic surgeons with extensive prior experience in performance of similar surgical procedures in nonpregnant subjects, the occurrence of this very unusual occurrence in our subject may emphasize the importance of ensuring adequate skill in undertaking this technically challenging procedure in pregnancy. Furthermore, the peculiarity of pregnancy and potential vulnerabilities that the unique anatomic and physiologic changes of pregnancy and delivery engenders in laparoscopic surgery for massive splenectomy at advanced gestations is underscored.

## 4. Conclusion

In symptomatic or severly hematologically compromised patients in whom treatment for hairy cell leukemia cannot be deferred till after delivery of the infant, laparoscopic splenectomy is an effective therapeutic option during the second trimester of pregnancy.
